# Publisher’s Notice

**Published:** 1830

**Authors:** 


PUBLISHERS NOTICE. We regret to learn that an impression has gone abroad, that Dr. Drake's association with the Jefferson Medical College, will interfere with his editorial duties. Not only will this not be the case, but his connexion with that institution will secure to the Journal a support that cannot fail to render its pages more interesting. We are authorized to state that Dr. D. has no idea of leaving Cincinnati. His associations of the past, and his prospects for the future, are all connected with the West, and will secure his permanent residence on this side of the mountains.

We have succeeded in getting out the first number of this volume within the time mentioned in our prospectus, and hope for the future to present it punctually to our subscribers. A similar punctuality on their part will afford the best evidence of their approbation, and most effectually secure our best exertions to gratify and instruct them.

PUBLISHERS.

